# Fraction of the T-Tubular Membrane as an Important Parameter in Cardiac Cellular Electrophysiology: A New Way of Estimation

**DOI:** 10.3389/fphys.2022.837239

**Published:** 2022-05-10

**Authors:** Olga Švecová, Markéta Bébarová, Milena Šimurdová, Jiří Šimurda

**Affiliations:** Department of Physiology, Faculty of Medicine, Masaryk University, Brno, Czechia

**Keywords:** sucrose, membrane capacitance, rat cardiomyocytes, new method, detubulation, t-tubules

## Abstract

The transverse-axial tubular system (t-tubules) plays an essential role in excitation-contraction coupling in cardiomyocytes. Its remodelling is associated with various cardiac diseases. Numerous attempts were made to analyse characteristics essential for proper understanding of the t-tubules and their impact on cardiac cell function in health and disease. The currently available methodical approaches related to the fraction of the t-tubular membrane area produce diverse data. The widely used detubulation techniques cause irreversible cell impairment, thus, distinct cell samples have to be used for estimation of t-tubular parameters in untreated and detubulated cells. Our proposed alternative method is reversible and allows repetitive estimation of the fraction of t-tubular membrane (*f*_t_) in cardiomyocytes using short-term perfusion of the measured cell with a low-conductive isotonic sucrose solution. It results in a substantial increase in the electrical resistance of t-tubular lumen, thus, electrically separating the surface and t-tubular membranes. Using the whole-cell patch-clamp measurement and the new approach in enzymatically isolated rat atrial and ventricular myocytes, a set of data was measured and evaluated. The analysis of the electrical equivalent circuit resulted in the establishment of criteria for excluding measurements in which perfusion with a low conductivity solution did not affect the entire cell surface. As expected, the final average *f*_t_ in ventricular myocytes (0.337 ± 0.017) was significantly higher than that in atrial myocytes (0.144 ± 0.015). The parameter *f*_t_ could be estimated repetitively in a particular cell (0.345 ± 0.021 and 0.347 ± 0.023 in ventricular myocytes during the first and second sucrose perfusion, respectively). The new method is fast, simple, and leaves the measured cell intact. It can be applied in the course of experiments for which it is useful to estimate both the surface and t-tubular capacitance/area in a particular cell.

## Introduction

The transverse-axial tubular system (t-tubules) plays an essential role in excitation-contraction coupling of skeletal and cardiac myocytes by spreading depolarization from the surface membrane to the vicinity of the terminal cisternae of the sarcoplasmic reticulum, the source of Ca^2+^ that trigger cellular contraction (for a recent review, see Setterberg et al., 2021).

The contribution of the t-tubules to cardiac cell function is crucially dependent on their characteristics, both structural and functional (Orchard et al., 2009; Pásek et al., 2012; Hrabcová et al., 2013; Hong and Shaw 2017; Smith et al., 2018). The t-tubular network is extremely dynamic and its remodelling, i.e., disruption or even loss of the t-tubules, has been demonstrated in a variety of cardiac diseases including ischemia, heart failure, and hypertension (e.g., Louch et al.,2004; Heinzel et al., 2008; Dibb et al., 2009; Ibrahim et al., 2011; Crossman et al., 2011; Wagner et al., 2012; Guo et al., 2013; Bryant et al., 2015; Crossman et al., 2017; Dibb et al., 2021). These changes in the t-tubules considerably affect the electrical and mechanical function of cardiomyocytes and contribute to further progression of the cardiac pathology.

Much effort has been made to analyse the t-tubular characteristics in cardiomyocytes. The first attempt to investigate the distribution of ionic channels between the surface and t-tubular membranes was based on the diffusion delay between the extracellular solution in the t-tubules and the bulk space after a rapid change in ionic concentrations (Shepherd and McDonough 1998). Over the last 10 years, substantially improved imaging methods have contributed significantly to a better understanding of the t-tubules properties in cardiomyocytes of healthy as well as failing/ischemic hearts (e.g., Crossman et al., 2011; Wagner et al., 2012; Guo et al., 2013; Guo and Song 2014; Rog-Zielinska et al., 2021). Recently, Uchida and Lopatin (2018) described changes in diffusion accessibility of cardiac t-tubules caused by hypoosmotic shock in cardiac myocytes. Using fluorescent dextran trapping and diffusion assay and computer modelling, they concluded that the t-tubular diameter irregularity (affected by constrictions and dilatations of t-tubules) was the major contributor to the diffusional and electrical properties of t-tubules.

The widespread detubulation techniques (e.g., Kawai et al., 1999; Brette et al., 2002; Komukai et al., 2002; Brette et al., 2006) make it possible to estimate the fraction of t-tubular membrane capacitance and other t-tubular characteristics such as ionic current densities using the formamide-induced osmotic shock and consequent rapid changes of cell volume and disconnection of t-tubules from the surface membrane. A certain disadvantage of the detubulation technique is the cell impairment by the irreversible process of physical detachment of the t-tubules which disables the paired statistical testing. The hard-to-determine fraction of the t-tubules that resist detubulation can impair measurement accuracy as well (Pásek et al., 2008a). Recently, a new method of detubulation using antidepressant imipramine has emerged that allows complete detubulation to be achieved (Bourcier et al., 2019).

We propose an alternative method for the evaluation of t-tubular characteristics namely the basic parameter—the fraction of t-tubular membrane capacitance/area. The theoretical basis of another version of this method has been preliminarily published as a preprint (Šimurda et al., 2021). The method is reversible and allows repeated measurements in the same cell under control conditions (Tyrode solution) and in the presence of isotonic sucrose solution. The substantial increase in electrical resistance of the t-tubular lumen allows for the electrical separation of the surface and t-tubular membranes in isolated atrial and ventricular myocytes.

## Materials and Methods

### Cell Isolation

Cardiomyocytes were isolated from atria and right ventricles of adult male Wistar rats (300 ± 20 g and 250 ± 50 g, respectively) anaesthetised by intramuscular administration of a mixture of tiletamine and zolazepam (65 mg kg^−1^; Zoletil^®^ 100 inj., *Virbac*, *France*), and xylazine (20 mg kg^−1^; Xylapan^®^ inj., *Spofa*, *Czech Republic*). The experiments were carried out with respect to recommendations of the European Community Guide for the Care and Use of Laboratory Animals; the experimental protocol was approved by the Local Committee for Animal Treatment at Masaryk University, Faculty of Medicine, and by the Ministry of Education, Youth and Sports (permission No. MSMT-29203/2012-30 and MSMT-33846/2017-3).

The dissociation procedure to obtain atrial and ventricular cardiomyocytes suitable for patch-clamp measurements was described in detail in our previous papers (e.g., Bébarová et al., 2005; Bébarová et al., 2016). In brief, the heart was retrogradely perfused *via* aorta with 0.9 mM CaCl_2_ Tyrode solution (3–5 min) and then with nominally Ca-free Tyrode solution (∼4.5 min). To isolate ventricular myocytes, the perfusion continued with the first digestion step (2.75 min), i.e., with nominally Ca-free Tyrode solution containing collagenase (type S, 0.2 mg/ml, *Yakult Pharmaceuticals*), protease (type XIV, *Sigma-Aldrich*; 0.053 mg/ml), and EGTA (*Sigma-Aldrich*; 34 μM). To isolate atrial myocytes, the first digestion step (3 min), was performed using Tyrode solution containing 0.6 μM CaCl_2_, collagenase (Roche A, 1 mg/ml, *Roche Diagnostics GmbH*), and protease (type XIV, *Sigma-Aldrich*; 0.053 mg/ml). In the second digestion step, the protease was omitted during the isolation of both atrial (14–24 min; median: 17 min) and ventricular myocytes (10–16 min; median: 13 min). The enzyme solution was then washed out in two steps by perfusion with the low calcium Tyrode solutions (0.09 and 0.18 mM CaCl_2_). All solutions were oxygenated with 100% O_2_ at 37°C.

Subsequently, the right and left auricles or the right ventricular free wall were dissected and minced in 0.18 mM CaCl_2_ Tyrode solution. After filtration through a nylon mesh, both atrial and ventricular isolated myocytes were exposed to gradually increasing external Ca^2+^ concentration (up to 0.9 mM within approx. 20 min).

### Solutions and Chemicals

Tyrode solution with the following composition was used both during the dissociation procedure and to perfuse myocytes during the measurements (in mM): NaCl 135, KCl 5.4, MgCl_2_ 0.9, HEPES 10, NaH_2_PO_4_ 0.33, and glucose 10 (pH was adjusted to 7.4 with NaOH). During measurements, 0.9 mM CaCl_2_ was added to the solution and CoCl_2_ (2 mM) was used for inhibition of I_Ca_. CoCl_2_ (*Sigma-Aldrich*) was prepared as 1 M stock solution in the deionized water. Sucrose (≥99.5%, *Sigma-Aldrich*) was dissolved in the deionized water to prepare the isotonic sucrose solution (0.3 M; osmolality 300 ± 5 mOsm/kg); 5 μM CaCl_2_ was added to maintain the membrane integrity and minimum conductivity. We have regularly checked the specific conductivity of the used distilled water (about 0.5–1.5 μS/cm). The final conductivity of the sucrose solution was ∼3.5 ± 0.2 μS/cm. The solutions were applied in close vicinity of the measured cell *via* a rapid perfusion system.

A sucrose solution containing 10, 25, and 100 μM BaCl_2_ (10 mM stock solution, BaCl_2_ dissolved in deionized water) was used to check the Ba^2+^-sensitive component of the membrane current during sucrose application.

To partially detubulate ventricular myocytes, the isolated cells were treated for 15 min with 75, 150, and 225 μM imipramine (according to Bourcier et al., 2019, but lower concentrations were used in our study to prevent the complete detubulation) and then they were centrifuged and the imipramine was washed using the control Tyrode solution.

The patch electrode filling solution contained (in mM): L-aspartic acid 130, KCl 25, MgCl_2_ 1, K_2_ATP 5, EGTA 1, HEPES 5, GTP 0.1, and Na_2_-phosphocreatine 3 (pH 7.25 adjusted with KOH).

### Electrophysiological Measurements and Evaluation

Single rod-shaped cells with well visible striations were used for recordings of the membrane current applying the whole-cell patch-clamp technique in the voltage-clamp mode. The patch pipettes were pulled from borosilicate glass capillary tubes and heat polished on a programmable horizontal puller (*Zeitz-Instrumente, Germany*). The resistance of the filled glass electrodes was below 1.5 MΩ to keep the access resistance as low as possible. For the generation of experimental protocols and data acquisition, the Axopatch 200B equipment and pCLAMP 9.2 software (*Molecular Devices*) were used. The measured ionic currents were digitally sampled at 200 kHz and stored on the hard disc. Experiments were performed at room temperature (23 ± 1°C). Experimental protocols are described in the Results.

### Mathematical Simulations

All calculations according to the formulas given in the Results section and in the Supplementary Material were performed using the computational software MATLAB v.7.2 (*MathWorks, Inc.*).

### Statistical Analysis

Evaluation of the data was performed using the computational software MATLAB R2020a (*MathWorks, Inc.*) except for the curve fitting that was performed using Clampfit 10.7 software (*Molecular Devices*). The results are presented as means ± S.E.M from *n* cells (Origin, version 2015, *OriginLab Corporation*, and GraphPad Prism, version 7.05, *GraphPad Software, Inc.*). The normality of the data distribution was tested using the Shapiro-Wilk test or Kolmogorov-Smirnov test (if *n* < 8). Paired and unpaired t-tests, and repeated measures ANOVA with the Bonferroni´s post-test were used to consider the statistical significance of the observed differences; *p* < 0.05 was considered statistically significant.

## Results

### A New Method for Repetitive Estimation of the Fraction of T-Tubular Membrane

**FIGURE 1:**
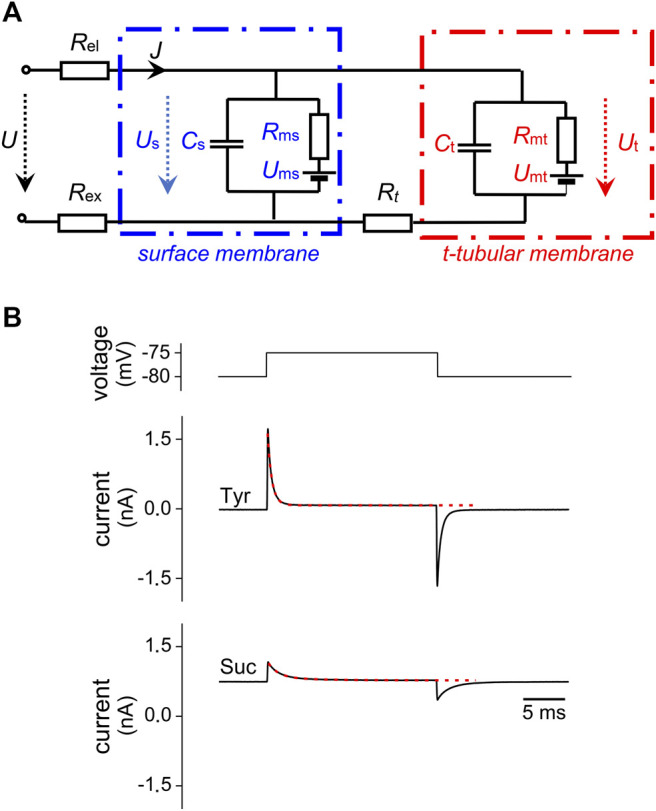
Principle of the new method. **(A)** Scheme of the electrical equivalent circuit of cardiomyocyte; *R*_el_—microelectrode resistance; *R*_ex_—extracellular resistance; *C*_s_, *R*_ms_, *U*_ms_, and *C*_t_, *R*_mt_, *U*_mt_—membrane capacitance, membrane resistance, reversal voltage of the surface and t-tubular membranes, respectively; *R*_t_—resistance of t-tubular lumen. The membrane resistances can be considered constant in the subthreshold range of membrane voltage. *U*, *U*_s_, and *U*_t_—the imposed voltage, surface membrane voltage, and t-tubular membrane voltage, respectively. *J*—the membrane current. **(B)** The imposed impulse of membrane voltage (upper panel) and representative current traces in the Tyrode (Tyr) and sucrose (Suc) solution including mono- and bi-exponential fits (red lines), respectively (middle and bottom panel).

The schematic diagram in Figure 1A represents the electrical equivalent circuit of a cardiac myocyte connected to a microelectrode. The surface membrane (capacitance *C*_s_, resistance *R*_ms_, and reversal voltage *U*_ms_) is separated from the t-tubular membrane (*C*_t_, *R*_mt_, and *U*_mt_) by longitudinal resistance of the t-tubular lumen (*R*_t_). The access resistance *R*_a_ = *R*_el_ + *R*_ex_ is composed of the microelectrode resistance *R*_el_ and resistance of extracellular medium *R*_ex_. The simple lumped equivalent circuit including the t-tubular system (similar to that applied for the same purpose by Cheng et al., 2011) was used. A model with distributed parameters of the t-tubular system of cardiomyocytes would be difficult to use for practical measurements (see the Discussion for more details).

The key idea of the proposed method is to achieve a significant increase in the electrical resistance of the t-tubular lumen *R*_t_ to electrically separate the surface and t-tubular membrane in whole-cell patch-clamp experiments. The low specific resistance of physiological solutions is associated with a low value of *R*_t_ which causes tight electrical coupling between the surface and t-tubular systems. Consequently, only the total membrane capacitance (marked *C*_Tyr_ in the following text) can be measured. Analysis of the electrical equivalent scheme describing cardiomyocyte in the whole-cell arrangement under conditions of a multiple increase in the resistance *R*_t_ allowed us to calculate the surface and t-tubular membrane capacitances (*C*_s_ and *C*_t_) separately as well as to estimate the fraction of t-tubular membrane *f*_t_ = *C*_t_/(*C*_s_ + *C*_t_). A sufficient increase in *R*_t_ was achieved by short-term perfusion of the measured cell with a low-conductive isotonic sucrose solution. The analysis was based on the resolution of two components in responses of membrane current to the imposed subthreshold voltage steps as shown in Figure 1B and in detail in Supplementary Figure S1. In the following, the symbols indicated in Figure 1A will refer exclusively to the values measured in the isotonic sucrose solution. *C*_m_ will indicate the total membrane capacitance estimated in the sucrose solution (*C*_m_ = *C*_s_ + *C*_t_) to distinguish it from *C*_Tyr_, measured in Tyrode solution.

**FIGURE 2:**
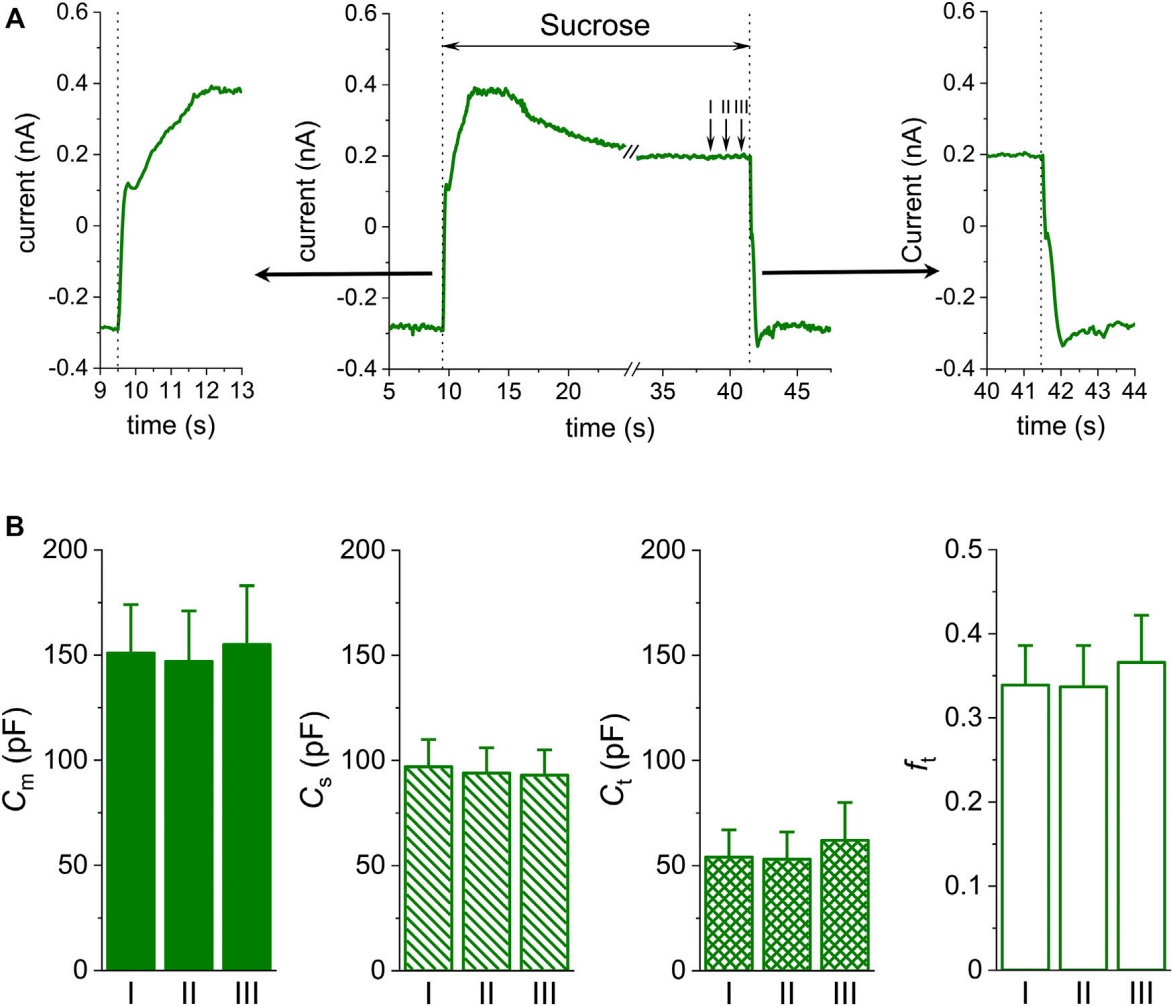
Time course of the sucrose effect. **(A)** Changes of the membrane current at −80 mV during application and subsequent wash-out of isotonic sucrose solution in a representative cell (middle panel, the time axis is interrupted at the interval when the voltage-ramp protocol was applied; left and right panels—details of 4 s of the recording shown in the middle panel at the time of start and end of sucrose application, respectively). **(B)** Average values of basic parameters evaluated in the presence of sucrose at the steady-state in three independent intervals I, II, and III at the end of sucrose application (*n* = 5, *p* > 0.05 within the same parameter; for the approximate position of the intervals I, II, and III, please see arrows in part **(A)**.

To get an idea of the processes that take place after the application of the sucrose solution, we recorded the time course of the current at the holding voltage −80 mV (Figure 2A). The current was reversed and surprisingly even increased in absolute value although the access resistance *R*_a_ increased substantially in the low conductivity medium, evidently due to an increased driving force. The membrane current is likely carried preferentially by K^+^ and Cl^−^ ions across the cell membrane. Considering the Nernst equations, the reversal voltages of all positive ions should acquire high negative values in contrast to a high positive value for Cl^−^. As a result, both K^+^ and Cl^−^ can flow out of the cell and help to maintain the electroneutrality of the media. The entry of ions from the sarcoplasm into the t-tubules can be expected to restrict the increase of *R*_t_. The evidence of a substantial role of the inward rectifying potassium current *I*_K1_ is given in the Discussion.

The broader theoretical basis of the new approach was preliminarily published as a preprint (Šimurda et al., 2021) which shows that the determination of *C*_t_ is associated with a certain inaccuracy, the limit of which can be calculated from the measured data. In the present work, a new way is followed to overcome the problem of *C*_t_ estimation. It is described in the Supplementary Material together with an outline of the derivation of calculation formulas. In the following text, the computational relationships used to determine the basic parameters of the electrical equivalent scheme (i.e., *C*_s_ and *C*_t_) supplemented by the calculation of *f*_t_ as an indicator of the area fraction occupied by the t-tubules will be listed.

The response of the capacitive current to subthreshold steps of membrane voltage (from the holding level *U*_1_ = −80 mV to optional *U*_2_) in the sucrose solution was approximated by a sum of two exponential functions and a constant (Supplementary Figure S1) in the Clampfit software (*Molecular Devices*). The resulting magnitudes of the exponential components (*J*_1_ and *J*_2_), corresponding time constants (*τ*_1_ and *τ*_2_), and steady-state current at the level *U*_2_ (*J*_∞,2_) were supplemented by the value of the steady-state current at the holding voltage (*J*_∞,1_). The values of *C*_t_, *C*_s_, *f*_t_, and the access resistance *R*_a_ could be calculated from the six parameters *J*_1_, *J*_2_, *τ*_1_, *τ*_2_, *J*_∞,1_, and *J*_∞,2_ using the following relationships derived in the Supplementary Material:

$$C_{\text{s}}=\frac{\tau_{\text{s}}}{R_{\text{a}}},\mathrm{where}\;\tau_{\text{s}}=\left(J_{1}+J_{2}-J_{\infty ,1}+J_{\infty ,2}\right)\frac{\tau_{1}\tau_{2}}{\tau_{1}J_{2}+\tau_{2}J_{1}}$$ (1)

$$\mathrm{and}\;\mathrm{the}\;\mathrm{access}\;\mathrm{resistance}\;R_{\text{a}}=\frac{U_{2}-U_{1}}{J_{1}+J_{2}-J_{\infty ,1}+J_{\infty ,2}}.$$ (2)

*C*_t_ is determined with an accuracy of ±4% in ventricular and ±5% in atrial cardiomyocytes (see the Supplementary Material for detailed explanation).

$$C_{\text{t}}∼\frac{\tau_{1}J_{2}+\tau_{2}J_{1}}{J_{1}+J_{2}}\frac{k_{c}}{R_{1}},\;\mathrm{where}\;R_{1}=\frac{R_{a}}{b-1},\;\mathrm{and}\;b=\frac{\tau_{1}^{2}J_{2}+\tau_{2}^{2}J_{1}}{\tau_{1}J_{2}+\tau_{2}J_{1}}\frac{\tau_{s}}{\tau_{1}\tau_{2}}.$$ (3)

The coefficient *k*_c_ was introduced as a correction for the mean error caused by the exchange of membrane resistances *R*_mt_ for *R*_ms_ in the approximate calculation of *C*_t_ as justified in the Supplementary Material (Eq. S18 and the accompanying text) and the value of *k*_c_ was set to 0.97 for ventricular and to 0.91 for atrial cardiomyocytes. The total membrane capacitance *C*_m_ and the fraction of t-tubular membrane *f*_t_ are expressed as

$$C_{\text{m}}=C_{\text{s}}+C_{\text{t}},f_{t}=\frac{C_{t}}{C_{m}}.$$ (4)

The experimental protocol consisted of a sequence of 300 rectangular voltage steps, 5 or 10 mV, 20 ms from the holding voltage of −80 mV applied at 25 Hz (a single step shown in Figure 1B, upper panel). This protocol was applied repeatedly if needed (max. 3 times). The representative current responses to a single voltage step in the Tyrode solution (Tyr) and in the isotonic sucrose solution (Suc) are illustrated in Figure 1B, middle and bottom panels, together with the mono-exponential fit in Tyr and bi-exponential fit in Suc (the red dashed lines). For details of evaluation of this representative cell, please see Supplementary Figure S1.

To obtain steady-state values of the evaluated parameters, the last 50 current responses before the sucrose wash-out were averaged and evaluated. No significant differences were observed among average values evaluated at three independent intervals at the end of sucrose application if the protocol was repeated until the steady-state current was reached (Figure 2B; *n* = 5, *p* > 0.05). However, the evaluated parameters were not significantly different even if being evaluated in ∼10 s from the start of the sucrose application which implies that reaching the steady-state of membrane current during sucrose application is not necessary to obtain steady-state values of the evaluated parameters (not illustrated).

### Accuracy of the Parameters Estimated Using the New Method

The accuracy of the method is critically dependent on the conditions ensuring that the entire cell surface is washed with sucrose solution or Tyrode solution during rinsing. The stream washing the cell must be carefully directed which is indicated by the ratio of access resistances measured in Tyrode and sucrose solution. For cells that have not been lifted from the bottom of the chamber, there is a risk that a part of the surface is not exposed to the solution. However, the violation of the conditions of correct measurement can be revealed from the parameters obtained by the bi-exponential approximation of the capacitive current. Incomplete solution exchange strongly affects the ratio of magnitudes and time constants of both components of the analysed part of the capacitive current. The component with the shorter time constant must be sufficiently expressed. Besides the ratio of the time constants and amplitudes of both capacitive current components, an additional quantity indicating an unacceptably low resistance of t-tubular lumens was the ratio of the resistance *R*_1_ defined in Eq. 3 and the resistance *R*_2_ that can be computed as

$$R_{2}=\frac{R_{a}}{R_{ms}||\left(R_{mt}+R_{t}\right)}=R_{a}\frac{J_{1}+J_{2}}{J_{\infty \_2}-J_{\infty \_1}}.$$ (5)

To decide whether a given measurement is acceptable and can be included in the overall evaluation, we applied the following criteria:

$$R_{a\_suc}-R_{a\_Tyr}>3M\Omega ,$$ (6a)

$$J_{2}/J_{1}>0.16,\tau_{2}/\tau_{1}>0.1,\mathrm{and}R_{1}/R_{2}>0.1.$$ (6b)

*R*_a_suc,_ and *R*_a_Tyr_ are access resistances in sucrose and Tyrode solution, *τ*_1_ and *J*_1_ refer to the component with the longer time constant, and *τ*_2_ and *J*_2_ to the component with the shorter time constant. In the following figures, only data from measurements fulfilling these criteria are included. For average values of *R*_a_suc,_ and *R*_a_Tyr_ in all selected cells, please see Supplementary Figure S2. Data selection based on the aforementioned criteria resulted in a decreased variability and, thus, in considerably increased accuracy of the resulting *f*_t_ values (Supplementary Figure S3).

### Comparison of the Average Values in Atrial and Ventricular Cardiomyocytes

**FIGURE 3:**
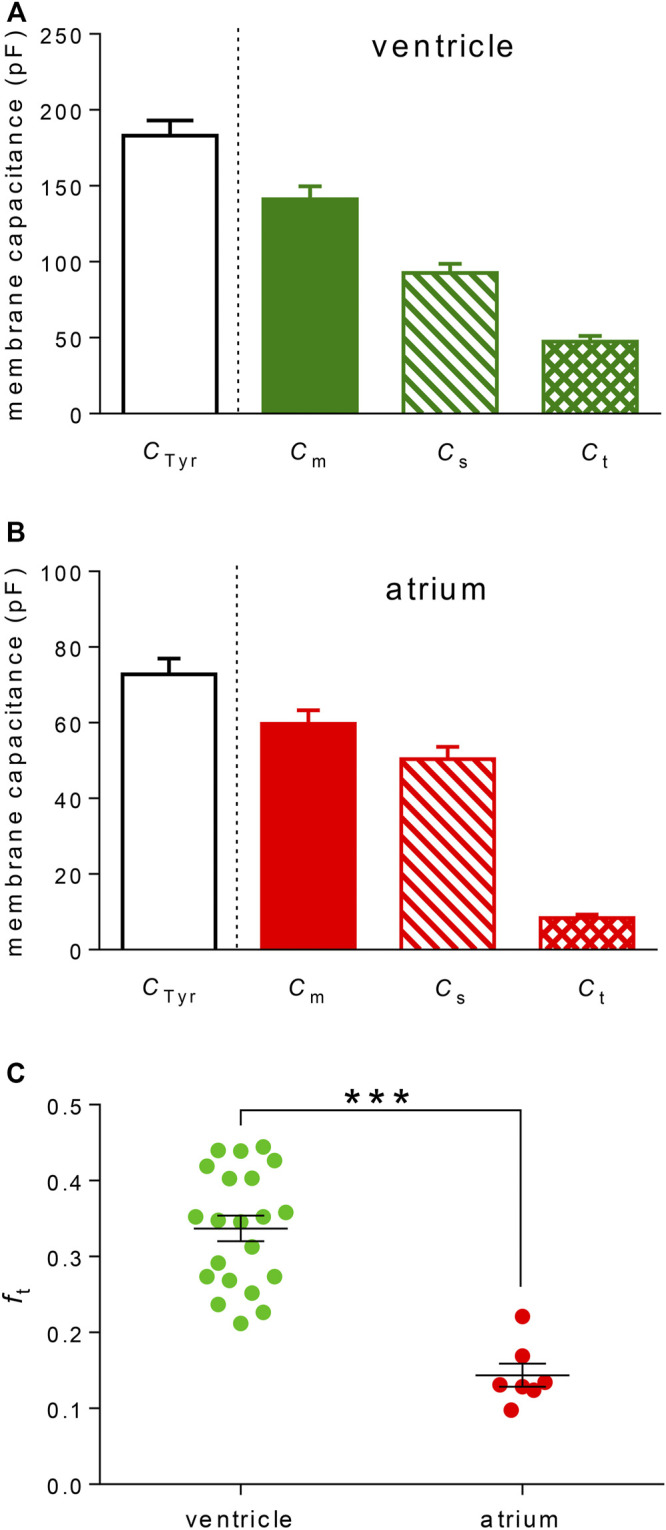
TATS characteristics evaluated using the newly developed method. **(A,B)** Average *C*_Tyr_, *C*_m_, *C*_s_, and *C*_t_ in ventricular **(A)** and atrial **(B)** cardiomyocytes. Values of all parameters in ventricular cells were significantly different at *p* < 0.001 (*n* = 21). In atrial cells, all parameters also significantly differed, but at various *P* (*p* < 0.05–0.001; *n* = 7). **(C)** The value of *f*_t_ was significantly lower in atrial vs. ventricular cardiomyocytes; ***—statistical significance at *p* < 0.001.

*C*_t_, *C*_s_, and *C*_m_ in the measured rat ventricular myocytes that passed the selection procedure described above were 47.3 ± 3.9, 92.7 ± 5.9, and 141.2 ± 8.6 pF on average, respectively (Figure 3A; *n* = 21). In rat atrial cardiomyocytes, *C*_t_, *C*_s_, and *C*_m_ were 8.4 ± 0.9, 50.4 ± 3.3, and 59.7 ± 3.6 pF on average, respectively, using the same selection criteria (Figure 3B; *n* = 7). It implies that *f*_t_ was significantly lower in rat atrial myocytes than that in rat ventricular myocytes (0.144 ± 0.015 vs*.* 0.337 ± 0.017; Figure 3C; *p* < 0.001) as may be expected considering the literary data and the substantially less developed t-tubular system in the atria. *C*_m_ evaluated from the data recorded during the sucrose application was significantly lower (by 23 and 18% in rat ventricular and atrial myocytes, respectively) than that evaluated in the control Tyrode solution *C*_Tyr_ (183.1 ± 9.9 pF in rat ventricular myocytes and 72.8 ± 4.2 pF in rat atrial myocytes; the respective *C*_m_ values—see above; *p* < 0.001 and 0.01 in rat ventricular and atrial myocytes, respectively; for a possible explanation, see the Discussion).

**FIGURE 4:**
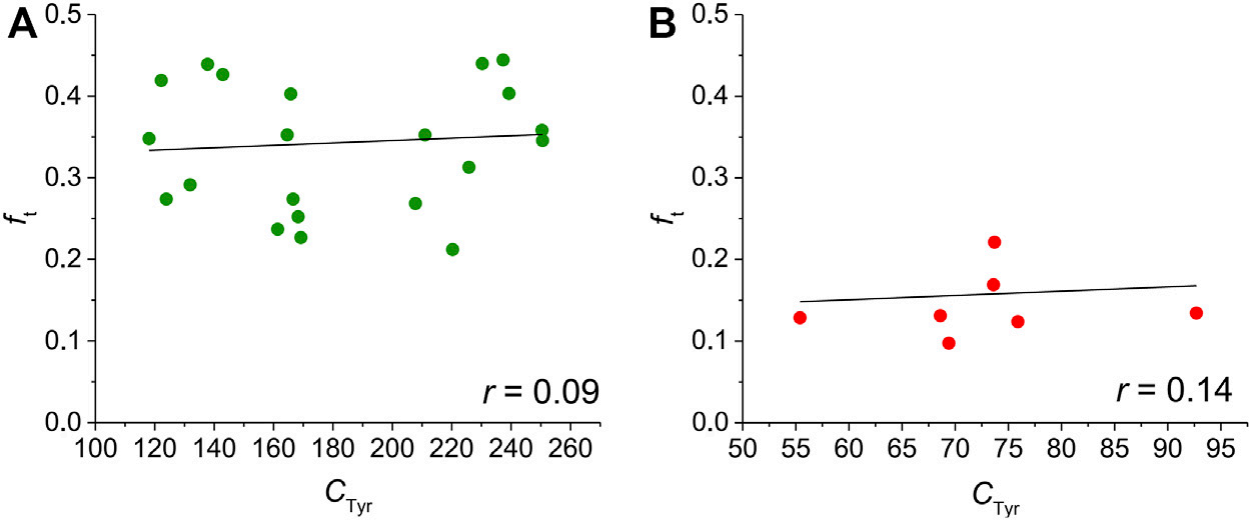
Correlation between *f*_t_ and *C*_Tyr_ in ventricular **(A)** and atrial **(B)** rat cardiomyocytes. No correlation can be observed in our data (*p* > 0.05 in both ventricular and atrial myocytes).

The value of *f*_t_ did not significantly correlate with *C*_Tyr_ (an indicator of the cell size) in both ventricular and atrial myocytes (Figure 4; for details, see the Discussion).

### Repetitive Estimation of the Parameters in a Single Cell

**FIGURE 5:**
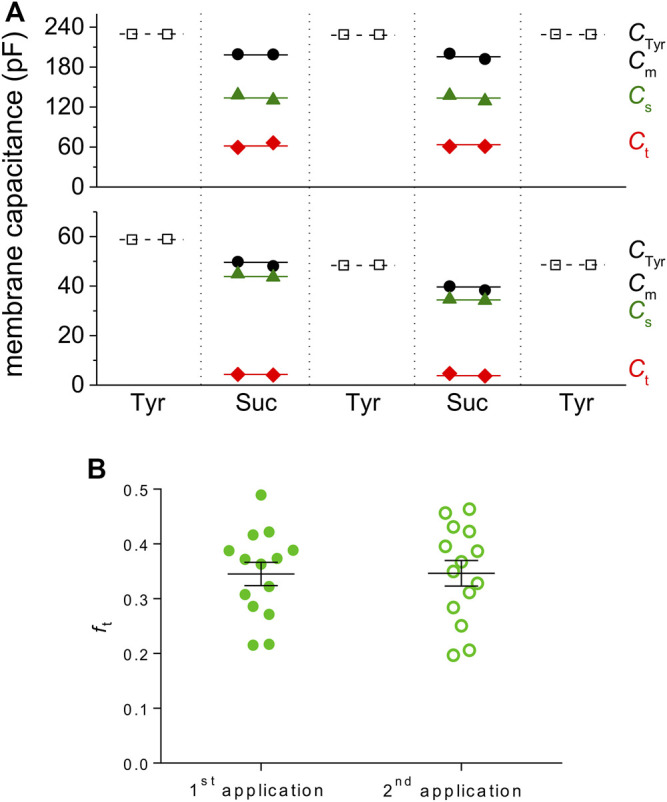
Repeated sucrose application allows evaluation of t-tubular characteristics several times in a particular cell. **(A)** Representative examples of two sucrose applications in a row in ventricular (upper graph) and atrial (lower graph) cardiomyocytes. The perfusion time with sucrose solution did not exceed 50 s. **(B)** The evaluated *f*_t_ was not different during subsequent sucrose applications (ventricular myocytes, *n* = 14, *p* > 0.05).

To test the most promising advantage of the new method, we tried to apply the sucrose solution twice in the same cell (both sucrose applications were separated by a wash-out period in Tyrode solution sufficient to recover control conditions; for representative examples of rat ventricular and atrial myocytes, Figure 5A). The average value of *f*_t_ was not different during the first and the second sucrose application (0.345 ± 0.021 and 0.347 ± 0.023, respectively; *n* = 14, *p* > 0.05; Figure 5B). Hence, our new method may be used several times in the same cell, enabling testing of dynamic changes of t-tubular parameters.

## Discussion

A new method for estimation of the fraction of t-tubular membrane (*f*_t_) in cardiomyocytes was developed, described, and experimentally proved in this study. Short-term perfusion with isotonic sucrose solution enabled to electrically separate the surface and t-tubular membranes. The most important advantage of this new method is its reversibility which allows repetitive measurements of *f*_t_ in the same cell.

### Verification of the Method and Justification of the Model

**FIGURE 6:**
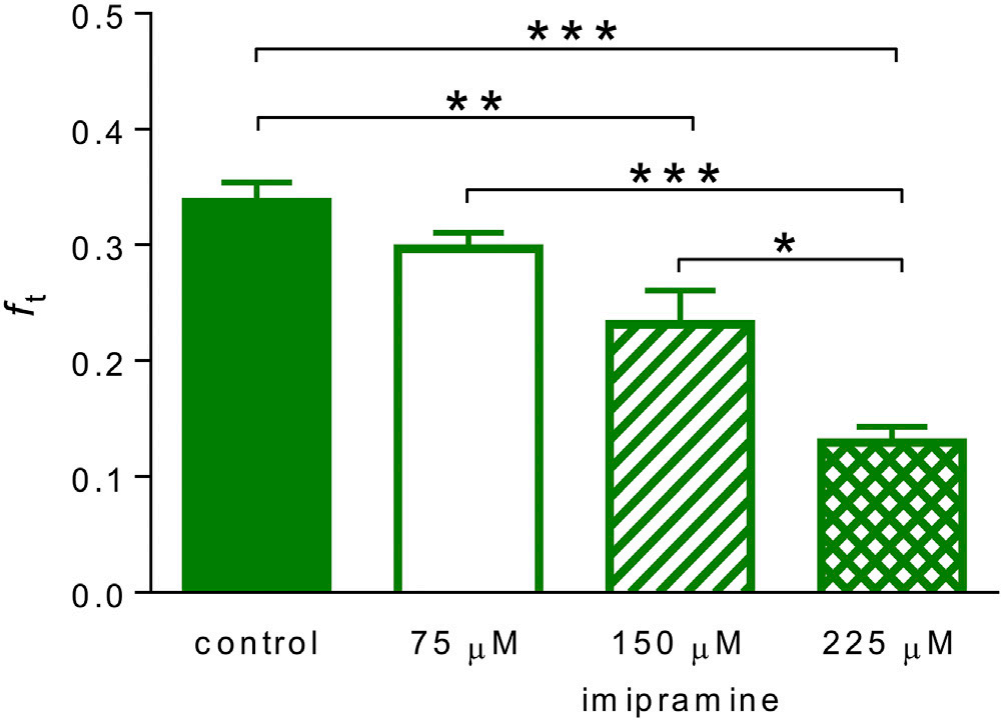
Partial detubulation revealed by the new technique. Comparison of the fraction of the t-tubular membrane *f*_t_ in control ventricular myocytes (*n* = 21) and in ventricular myocytes partially detubulated using 75, 150, and 225 μM imipramine (*n* = 5, 8, and 8, respectively); *, **, and ***—statistical significance at *p* < 0.05, 0.01, and 0.001, respectively.

To verify our method, we investigated the effect of detubulation induced by imipramine (Bourcier et al., 2019). We first confirmed the detubulation effect of imipramine by measuring the inward rectifier potassium tail current (*I*_K1,tail_) in Tyrode solution in both control ventricular myocytes and in ventricular myocytes pre-treated with 225 μM imipramine in which *I*_K1,tail_ was strongly suppressed, as expected (Supplementary Figure S4A). As can be seen in Figure 6, the t-tubular membrane fraction *f*_t_ determined by our method was significantly reduced in partially detubulated ventricular cardiomyocytes in a concentration-dependent manner. At the complete detubulation, the determination of *f*_t_ fails because the method is based on the analysis of two clearly distinguishable exponential components [indicated by conditions (6b)].

A number of quantitative models have been published so far aimed at describing the electrical properties of the tubular system in skeletal muscle cells (Adrian et al., 1969; Eisenberg et al., 1972; Mathias et al., 1977; Penderson et al., 2011). These models with distributed parameters follow the results of cable theory. They are usually formulated in such a general way that they can also be applied to cardiac cells, but hardly for practical measurement of tubular membrane capacitance. Mathias et al. (1977) developed and described in detail a model (called mesh model) based on an analysis of a random network of miniature cables connecting nodes. The whole system is described by a combination of differential and difference equations. Depending on the ratio between the length constant λ and the radius *a* of the cell (approximately cylindrical), the solution of the system can be divided into several areas. Assuming λ >> a, a lumped approximation of the tubular system is justified in a form corresponding to our simple model, created by the luminal resistance of the tubular system in series with a parallel combination of membrane resistance and capacitance. According to the recent study of Scardigli et al. (2017), tubular length constant *λ* = 290 ± 90 μm and *a ∼* 13 μm in rat ventricular myocytes resulting in *λ*/*a* ∼ 22. This ratio may be however altered under conditions of sucrose solution. Both resistances determining *λ* will be increased, however, the actual change can only be roughly estimated. Even if, in the extreme case, the luminal resistance per unit length of the tubule increased 100 times more than the resistance of the membrane, the condition λ > r would still be maintained. Lumped models of electrical properties of cardiomyocytes, including the tubular system, have already been used in other studies (Pásek et al., 2006; Pásek et al., 2008c; Cheng et al., 2011).

### Origin of the Membrane Current in Sucrose

**FIGURE 7:**
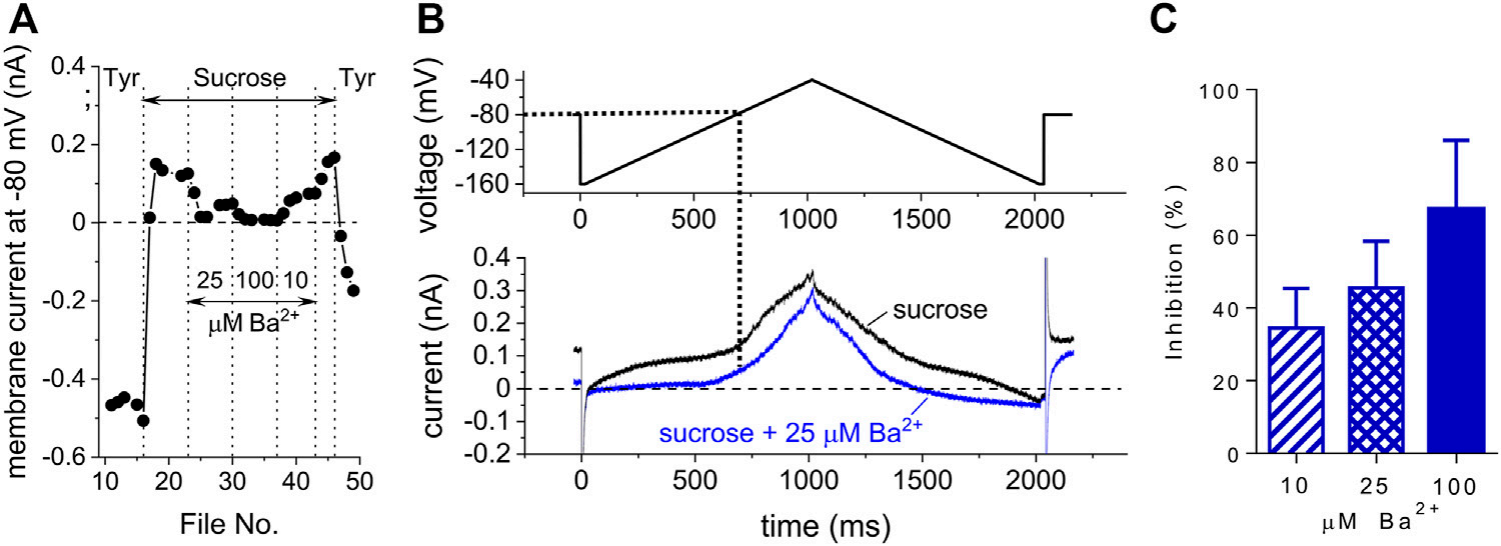
Effect of Ba^2+^ on the membrane current in the presence of sucrose. **(A)** Time course of changes of the membrane current at −80 mV in a representative cell during consecutive application of sucrose alone and in combination with 10, 25, and 100 μM Ba^2+^. **(B)** Changes of the membrane current during a ramp stimulation (lower panel; for the stimulation protocol, see the upper panel). **(C)** Average inhibition of the membrane current at −80 mV in sucrose at 10, 25, and 100 μM Ba^2+^ (*n* = 3).

Considering the minute content of ions in the isotonic sucrose solution (supplemented only with 5 μM CaCl_2_, the conductivity of ∼3.5 μS/cm), the origin of the membrane current in sucrose is not entirely clear. The most likely candidate of the flowing current is inward rectifier potassium current *I*_K1_ which can be partially opposed by a negative chloride current with a high positive equilibrium voltage. We tested the effect of a specific *I*_K1_ inhibitor Ba^2+^ in three selected concentrations 10, 25, and 100 μM. The membrane current in sucrose was inhibited by 34, 46, and 67%, respectively (Figure 7). It implies that *I*_K1_ is the predominant component of the membrane current, especially around the holding voltage −80 mV. We tried to further support this result by measuring the effect of Ba^2+^ on *I*_K1,tail_ induced by transient accumulation of t-tubular potassium ions (Cheng et al., 2011; Moench et al., 2013). However, this intention failed because *I*_K1_,_tail_ was suppressed during application of the sucrose solution (Supplementary Figure S4B), very likely due to a high negative reversal voltage (see below) which prevented appearance of the inward current.

In sucrose, the resting membrane voltage considerably dropped to about −140 mV (Figure 7B), thus, the driving force of the ionic current was markedly enhanced. This can explain the large sucrose-induced outward current in Figure 2A. The shift of the resting voltage in sucrose solution corresponds well with the observations of Bouchard et al. (2004) who indicated a substantial shift of reversal voltage and decrease of slope conductance at low extracellular K^+^ concentrations. Figure 7 provides yet further evidence supporting the idea that *I*_K1_ is a major component of the ionic current in sucrose solution. Due to the effect of Ba^2+^, the negative reversal voltage dropped significantly to a level around −100 mV, as can be seen clearly from the current response to the descending part of the imposed voltage ramp.

### The Fraction of T-Tubular Membrane *f*_t_ Estimated by Various Techniques

Marked differences can be observed in *f*_t_ values evaluated using various techniques in ventricular myocytes, even in those from healthy hearts. Using the diffusion technique and measurements of whole-cell capacitance and cell dimensions, Shepherd and McDonough (1998) suggested *f*_t_ of 0.56 in guinea-pig ventricular myocytes, which is consistent with data acquired by imaging techniques in rat, guinea-pig, and human ventricular myocytes (over 0.5; Amsellem et al., 1995; Soeller and Cannell 1999; Ohler et al., 2009).

In contrast, studies using the often-used detubulation techniques report lower values of *f*_t_ in rat ventricular myocytes. Most data were obtained using hyperosmotic shock (e.g., *f*_t_ = 0.264 in Kawai et al., 1999, 0.29 in Brette and Orchard 2006, 0.32 in Brette et al., 2006, 0.315 in Bryant et al., 2015); later studies report values obtained from hypoosmotic shock (0.27 in Moench et al., 2013) or from the effect of imipramine (∼0.4 in Bourcier et al., 2019). The value *f*_t_ = 0.337 ± 0.017 estimated by our new method (Figure 3C) fits well with the range of values obtained so far by detubulation approaches. Considering all these data and despite attempts to explain the differences (Pásek et al., 2008b), the true value of *f*_t_ in ventricular myocytes is still unclear, estimated somewhere between ∼0.25 and 0.55.

The published data on t-tubular characteristics in atrial myocytes are even more diverse and rarer than that in ventricular cells. The t-tubules were identified by imaging techniques in atrial myocytes of various species including rat and human (e.g., Dibb et al., 2009; Wakili et al., 2010; Richards et al., 2011; Frisk et al., 2014; Glukhov et al., 2015; Yue et al., 2017). Yue et al. (2017) found out that identifiable t-tubules may be detected in more than 80% of isolated mouse atrial myocytes. However, only a minority of atrial myocytes contain well-organized t-tubules in small rodents, e.g., in the rat (∼10%, Frisk et al., 2014). As expected, the value *f*_t_ estimated by our new method was significantly lower in rat atrial myocytes in comparison to that in rat ventricular myocytes (Figure 3C). Considering almost zero t-tubular area estimated by Caldwell et al. (2014) in the rat atria (using di-4-ANEPPS membrane staining), we were surprised by our relatively high atrial *f*_t_ value (0.144 ± 0.015). Our results agreed rather with the data published by Yue et al. (2017) who estimated the t-tubular area between 0 and 24% in mouse atrial myocytes (∼10% on average in male mice using di-4-ANEPPS membrane staining). To our knowledge, the only study using formamide detubulation in atrial cells (Brette et al., 2002) suggests a value of *f*_t_ ∼ 0.06. The heterogeneity of the tubular system in the atria is apparently enormous (Frisk et al., 2014).

In addition to various measurement techniques, interspecies differences and heterogeneity of t-tubular density in both ventricular and atrial myocytes are likely the main cause of the different results (Richards et al., 2011; Caldwell et al., 2014; Frisk et al., 2014; Yue et al., 2017).

### Does the Content of T-Tubules Correlate With Cell Size?

In our data, we did not observe a significant correlation between *f*_t_ and size of the cells (estimated as *C*_Tyr_) in both rat ventricular and atrial myocytes (Figure 4). Regarding ventricular myocytes, this finding is not surprising because previous studies documented overall well-developed t-tubules in the investigated ventricular myocytes of various species including rat (e.g., Soeller and Cannell 1999; Dibb et al., 2009; Richards et al., 2011; Jayasinghe et al., 2012; Frisk et al., 2014). Therefore, no correlation between the cell size and t-tubular content may be expected and was not observed in previous studies (e.g., Richards et al., 2011; Frisk et al., 2014).

Controversial information may be found regarding t-tubular content in atrial cells of various sizes. Frisk et al. (2014) showed that the size of rat atrial myocytes without and with either disorganized or organized t-tubules did not differ (Figure 3D in Frisk et al., 2014) which agrees well with our data (Figure 4B). In contrast, the proportion of rat atrial myocytes with the t-tubules was higher in the wider cells in the study by Smyrnias et al. (2010); imaging identification of t-tubules). Similarly, the t-tubular content correlated with the cell width in mouse atrial myocytes (Yue et al., 2017; imaging identification of t-tubules). We missed this correlation in our data (Figure 4B) which may be related to the fact that we likely unintentionally selected only wider cells for our patch-clamp measurements.

More developed t-tubules could be found in wider myocytes isolated from the atria of big mammals, such as dog, horse, cow, and sheep (Wakili et al., 2010; Richards et al., 2011). As discussed by Richards et al. (2011), the difference between atrial myocytes of small rodents (rat) and big mammals may be related to the lower width of atrial myocytes of small rodents (the width of about 10 μm in rat, e.g., Dibb et al., 2009; Walden et al., 2009) and to the necessity of more developed t-tubules in wider cardiomyocytes to ensure the synchronized rise of Ca^2+^ in the whole cardiomyocyte and, thus, its synchronized contraction (reviewed by Dibb et al., 2013).

### Advantages and Limitations of the New Method

As mentioned above, the most important advantage of the new method is its reversibility. It allows to directly compare parameters of the total cell membrane measured in the control Tyrode solution and the t-tubular membrane measured in the sucrose solution in the same cell. Thus, paired comparison of the differences may be done which is the preferable way of evaluation whenever it is possible. Since the estimated *f*_t_ did not differ during the first and second sucrose application in the same cells (Figure 5), the method may be used even for analysis of short-term changes in structure and function of the t-tubular system, induced by e.g., a transient hypoosmotic state (Moench et al., 2013) which may be relevant for the clinical practice (e.g., Kanda et al., 2004; Silveira et al., 2018). Both the paired statistical testing and analysis of short-term changes of the t-tubules are impossible when the usually used detubulation method is applied. We also plan to extend the use of the method to the estimation of other parameters characterizing the t-tubular membrane, such as the fraction of various ionic currents in the t-tubules because literary data are sparse and diverse in some cases.

A certain limitation of the method is the fact that the set of parameters obtained by the bi-exponential approximation of the capacitive current recorded in the sucrose solution does not allow an accurate calculation of all elements of the electrical equivalent circuit (Figure 1A). In our preliminary study (Šimurda et al., 2021), this problem was solved by an additional assumption of direct proportionality between the ratio of membrane conductivities and the ratio of membrane capacitances measured in t-tubular and surface membranes. In this work, we introduced a simpler way to estimate the t-tubular membrane capacitance *C*_t_ ± SD with the accuracy limited by the given theory. In ventricular and atrial cardiomyocytes, the value of SD was 4% and 5%, respectively, as justified in the Supplementary Material.

The accurate measurements require a reliable washing of the entire cell surface with the selected solution. A violation of this condition is reflected in the set of parameters obtained by the bi-exponential approximation of the capacitive current. The condition (Eq. 6a) is to guarantee that the source of the perfusion solution is not too far from the measured cell. The remaining conditions (Eq. 6b) indicate that all parts of the cell are sufficiently washed. Unfortunately, many measured data were discarded (Supplementary Figure S3). Considering the fact that many measurements were discarded due to violation of conditions (Eq. 6b), we expect that the number of well-measured cells may be increased by lifting the measured cell slightly. It well agrees with the findings recently published by Uchida and Lopatin (2018) who emphasize the necessity of lifting the measured cell up to ensure proper t-tubular diffusion with the applied solution. It is a risky procedure, but it may help improve the diffusion of sucrose into the t-tubular system and, thus, increase the yield of our newly proposed method.

Another limitation may result from the fact that sucrose application seems to affect the estimated value of the cell membrane capacitance. *C*_m_ evaluated in the sucrose solution was significantly lower than *C*_Tyr_ evaluated in the Tyrode solution (Figures 3A,B). It is in agreement with the study by Vaughan et al. (1972) on skeletal muscle fibers. They showed that the cell membrane capacitance may be reduced if the cell is exposed to a solution with low content of ions. Anyway, the value of *f*_t_ should not be affected by this side effect of sucrose application if the surface and t-tubular membranes are affected evenly by sucrose. In addition, the actual values of *C*_s_ and *C*_t_ can be estimated by multiplying by the *C*_Tyr_/*C*_m_ coefficient.

## Conclusion

We have developed a new approach to the determination of the fraction of t-tubular membrane based on the perfusion of the measured cell with a low-conductivity isotonic solution. Its advantage over existing methods is the possibility of repeated measurements on the same cardiomyocyte and thus paired statistical testing. In the next work, we assume its extension to the measurement of t-tubular ionic current fractions. The method can be useful for studying short-term changes in the t-tubular system.

## Data Availability

The raw data supporting the conclusion of this article will be made available by the authors, without undue reservation.

## Abbreviations

All symbols refer to the measurement in sucrose solution unless otherwise stated: *C*_m_, Total membrane capacitance; *C*_t_, Capacitance of t-tubular membrane; *C*_Tyr_, Total membrane capacitance measured in Tyrode solution; *C*_s_, Capacitance of surface membrane; *f*_t_, Fraction of t-tubular membrane; *I*_K1,tail_, Inward rectifier potassium tail current; *J*, Membrane current; *J*_1_, *J*_2_, Magnitudes of exponential components of the capacitive current; *J*_∞,1_, *J*_∞,2_, Steady-state currents at the membrane voltage *U*_1_ and *U*_2_; *k*_c_, Correction factor related to *C*_t_; *R*_a_, Access resistance; *R*_a_suc_, *R*_a_Tyr_, Access resistance in sucrose and Tyrode solution; *R*_el_, Microelectrode resistance; *R*_ex_, Resistance of the extracellular medium; *R*_1_, *R*_2_, Auxiliary directly quantifiable resistances; *R*_ms_, *R*_mt_, Resistances of surface and t-tubular membrane; *R*_t_, Total resistance of t-tubular lumen; *τ*_1_, *τ*_2_, Time constants of exponential components of the capacitive current; *U*, Membrane voltage; *U*_1_, *U*_2_, Imposed levels of membrane voltage; *U*_s_, *U*_t_, Membrane voltage on the surface and t-tubular membrane; *U*_ms_, *U*_mt_, Resting voltage of the surface and t-tubular membranes.
