# Supplementary material for: Fraction of the T-Tubular Membrane as an Important Parameter in Cardiac Cellular Electrophysiology: A New Way of Estimation
Source: Front Physiol. 2022 May 10;13:837239. doi: 10.3389/fphys.2022.837239 (PMC9127156; doi:10.3389/fphys.2022.837239)
Supplement: Supplementary file 1 [file DataSheet1.PDF]

FRACTION OF THE T-TUBULAR MEMBRANE AS AN IMPORTANT PARAMETER IN CARDIAC CELLULAR ELECTROPHYSIOLOGY: A NEW WAY OF ESTIMATION

Olga Švecová, Markéta Bébarová, Milena Šimurdová, Jiří Šimurda

Supplementary Material

Theoretical background. Derivation of the expressions to calculate capacitances of the surface and tubular membranes

The lumped equivalent circuit of cardiac cell membranes (Fig. 1A in the main text) is valid in the subthreshold range of membrane voltage. The scheme includes separately membrane properties of the surface and tubular membranes. It is described mathematically by a system of two differential equations for the time-dependent variables, membrane voltages U_s and U_t (for more details see Šimurda *et al.* 2021). These equations can be solved following the standard approach to systems of linear non-homogeneous differential equations. In particular, the response to the imposed subthreshold step of membrane voltage U from the level U_1 to U_2 leads to a sum of two exponential functions

$$U_s(t) = c_1 e^{-\frac{t}{\tau_1}} + c_2 e^{-\frac{t}{\tau_2}} + \frac{k_t k_{Us} + k_{Ut} + k_t U_2}{k_{st} k_t - k_{et}}, \tag{S1}$$

$$U_t(t) = \frac{c_1}{k_t - \frac{\tau_t}{\tau_1}} e^{-\frac{t}{\tau_1}} + \frac{c_2}{k_t - \frac{\tau_t}{\tau_2}} e^{-\frac{t}{\tau_2}} + \frac{k_{Us} + k_{Ut} k_{Us} + U_2}{k_{st} k_t - k_{et}},$$

where

$$k_t = 1 + \frac{R_t}{R_{mt}}, \quad k_{st} = 1 + \frac{R_a}{R_{ms}} + \frac{R_a}{R_t}, \quad k_{et} = \frac{R_a}{R_t}, \quad k_{Ut} = U_{mt} \frac{R_t}{R_{mt}}, \quad k_{Us} = U_{ms} \frac{R_a}{R_{ms}}, \tag{S2}$$

$$\tau_s = R_a C_s, \quad \tau_t = R_t C_t.$$

Taking into account initial conditions, the constants c_1 and c_2 are expressed as

$$c_1 = \frac{U_1 - U_2}{k_{st} k_t - k_{et}} \frac{\tau_1 k_t - \tau_t}{\tau_1 - \tau_2}, \quad c_2 = \frac{U_1 - U_2}{k_{st} k_t - k_{et}} \frac{\tau_t - \tau_2 k_t}{\tau_1 - \tau_2}. \tag{S3}$$

The time constants τ_1 and τ_2 of the two exponential terms can be assessed from properties of the roots of the characteristic equation

$$\frac{k_t}{\tau_t}+\frac{k_{st}}{\tau_s}=\frac{1}{\tau_1}+\frac{1}{\tau_2}, \quad \frac{k_{st}k_t-1}{\tau_s\tau_t}=\frac{1}{\tau_1\tau_2}. \tag{S4}$$

The only measured quantity is membrane current *J*, which is simply related to membrane voltage U_s by the Ohm law

$$J=\frac{U-U_s}{R_a}. \tag{S5}$$

Considering the equations (S1) and (S5), the response of current *J* to a step of membrane voltage ($U_1 \rightarrow U_2$) can be expressed as a sum of two exponential terms and a constant

$$J=J_1 e^{-\frac{t}{\tau_1}}+J_2 e^{-\frac{t}{\tau_2}}+J_{\infty,2}.$$

$J_{\infty,2}$ is the steady-state current at the voltage U_2. The value $J_{\infty,1}$ at U_1 corresponds with the initial condition.

The bi-exponential approximation of the recorded capacitive current provides numeric values of six parameters, namely J_1, J_2, $J_{\infty,1}$, $J_{\infty,2}$, τ_1, and τ_2. Their dependence on the elements of the lumped equivalent circuit in Fig. 1A is given by a set of six equations

$$J_1=\frac{U_2-U_1}{R_a}\frac{1}{k_{st}k_t-k_{et}}\frac{\tau_1 k_t-\tau_t}{\tau_1-\tau_2}, \quad J_2=\frac{U_2-U_1}{R_a}\frac{1}{k_{st}k_t-k_{et}}\frac{\tau_t-\tau_2 k_t}{\tau_1-\tau_2},$$

$$J_{\infty,1}=\frac{U_1\left(k_{st}k_t-k_{et}-k_t\right)-k_t k_{Us}-k_{et}k_{Ut}}{R_a\left(k_{st}k_t-k_{et}\right)}, \quad J_{\infty,2}=\frac{U_2\left(k_{st}k_t-k_{et}-k_t\right)-k_t k_{Us}-k_{et}k_{Ut}}{R_a\left(k_{st}k_t-k_{et}\right)}, \tag{S6}$$

$$\frac{1}{\tau_1}+\frac{1}{\tau_2}=\frac{k_t}{\tau_t}+\frac{k_{st}}{\tau_s}, \quad \frac{1}{\tau_1\tau_2}=\frac{k_{st}k_t-k_{et}}{\tau_s\tau_t}.$$

The number of six parameters estimated from records of *J* is not sufficient to calculate the values of all eight electrical elements of the equivalent circuit, *i.e.*, C_s, C_t, R_a, R_t, R_{ms}, R_{mt}, U_{ms}, and U_{mt}. This study is however focused on the assessment of capacitances C_s and C_t of the surface and tubular membranes.

Expressing the sum $J_1 + J_2 - J_{\infty,1} + J_{\infty,2}$ from (S6), we arrive at a formula for calculating the access resistance $R_a = R_{el} + R_{ex}$

$$R_a=\frac{U_2-U_1}{J_1+J_2-J_{\infty,1}+J_{\infty,2}}. \tag{S7}$$

According to the last equation in (S6)

$$\tau_s = \frac{\left(k_{st}k_t - k_{et}\right)}{k_t}\frac{k_t}{\tau_t}\tau_1\tau_2, \tag{S8}$$

Expressing $J_1 + J_2$ and J_1/J_2 from (S6), and considering definitions of the symbols (S2), we get

$$\frac{k_{st}k_t - k_{et}}{k_t} = 1 + \frac{R_a}{R_{ms} \,||\left(R_{mt} + R_t\right)}, \tag{S9}$$

$$\frac{k_t}{\tau_t} = \frac{1}{C_t\left(R_t \,||\, R_{mt}\right)} = \frac{J_1 + J_2}{\tau_1 J_2 + \tau_2 J_1}. \tag{S10}$$

For convenience, parallel combinations of the resistances in (S9) and (S10) are expressed by the symbol || (this means for example $R_t \,||\, R_{mt} = R_t R_{mt} / (R_t + R_{mt})$ and this symbol will continue to be used). Substituting of (S9) and (S10) into (S8) and considering (S7), the time constant $\tau_s = R_a C_s$ can be calculated as a function of the measured parameters

$$\tau_s = \left(J_1 + J_2 - J_{\infty,1} + J_{\infty,2}\right)\frac{\tau_1\tau_2}{\tau_1 J_2 + \tau_2 J_1}.$$

The capacitance of the surface membrane can then be expressed exactly:

$$C_s = \frac{\tau_s}{R_a}. \tag{S11}$$

Similarly, the value of C_t might be obtained as $C_t = \tau_t / R_t$. However, we were not able to express the ratio τ_t / R_t as a function of the measured parameters. Another possibility was to express C_t from (S10).

However, even ($R_{mt} \,||\, R_t$) could not be expressed directly from the measured parameters in contrast to parallel combination ($R_{ms} \,||\, R_t$).

$$R_{ms} \,||\, R_t = \frac{R_{ms}R_t}{R_{ms} + R_t} = \frac{R_a}{k_{st} - 1}, \quad where \quad k_{st} = \frac{\tau_1^2 J_2 + \tau_2^2 J_1}{\tau_1 J_2 + \tau_2 J_1}\frac{\tau_s}{\tau_1\tau_2}. \tag{S12}$$

The equation (S12) follows directly from the definition of k_{st} in (S2) and k_{st} can be expressed from the penultimate equation in (S6) after substitution for k_t / τ_t from (S10).

If $R_t << R_{mt}$ or $R_{mt} / R_{ms} \rightarrow 1$, the parallel combination $R_{mt} \,||\, R_t$ can be approximately replaced by $R_{ms} \,||\, R_t$. Another version of this method described in Šimurda *et al.* (2021) allows determining (for a given measurement) of the numerical values of all resistances in the electrical equivalent circuit of the cell. Results from both ventricular and atrial cardiomyocytes suggest that the substitution of $R_{ms} \,||\, R_t$ for $R_{mt} \,||\, R_t$ can be used with the accuracy limitation derived below.

Solution of equation (S10) for C_t after substitution of $R_{ms} \parallel R_t$ for $R_{mt} \parallel R_t$ finally gives

$$C_t \cong \frac{\tau_1 J_2 + \tau_2 J_1}{J_1 + J_2} \frac{1}{R_t \parallel R_{ms}}. \tag{S13}$$

The resistance $R_{ms} \parallel R_t$ is given by equation (S12).

Accuracy of C_t measurements

Let C_{t_e} be the exact values and C_{t_a} approximate values of C_t.

$$C_{t\_e} = \frac{\tau_1 J_2 + \tau_2 J_1}{J_1 + J_2} \frac{1}{R_t \parallel R_{mt}}, \qquad C_{t\_a} = \frac{\tau_1 J_2 + \tau_2 J_1}{J_1 + J_2} \frac{1}{R_t \parallel R_{ms}}. \tag{S14}$$

Let us further introduce relative quantities r_t and r_{mst}

$$r_t = \frac{R_t}{R_{mt}} \quad \text{and} \quad r_{mst} = \frac{R_{ms}}{R_{mt}}. \tag{S15}$$

To assess the error, it is necessary to express the ratio C_{t_a} / C_{t_e}

$$\frac{C_{t\_a}}{C_{t\_et}} = \frac{\dfrac{R_t + R_{ms}}{R_t R_{ms}}}{\dfrac{R_t + R_{mt}}{R_t R_{mt}}} = \frac{1}{r_{mst}} \frac{r_t + r_{mst}}{1 + r_t}. \tag{S16}$$

The error ε can be expressed as

$$\varepsilon = \frac{C_{t\_a} - C_{t\_e}}{C_{t\_e}} = \frac{r_t \left(1 - r_{mst}\right)}{r_{mst} \left(1 + r_t\right)} \tag{S17}$$

and the relation between the exact and the approximated tubular capacitance as

$$C_{t\_e} = \frac{C_{t\_a}}{1 + \varepsilon}. \tag{S18}$$

The error ε was calculated for all available ventricular and atrial cells according to (S17). For almost all measurements $\varepsilon > 0$, because $R_{mt} > R_{ms}$. The mean value $\varepsilon_{\text{mean}}$ was 0.03 in ventricular ($n = 21$) and 0.09 in atrial ($n = 7$) cardiomyocytes. Thus, the correction coefficient introduced in equation (3) was set to $k_c = 1 - 0.03 = 0.97$ in ventricular and $k_c = 1 - 0.09 = 0.91$ in atrial cells.

In atrial cells, where the t-tubular system is less developed, the difference $R_{mt} - R_{ms}$ and $\varepsilon_{\text{mean}}$ were greater. The standard deviation in percent was ±4% and ±5% in ventricular and atrial cells, respectively.

Verification of the derived computational formulas

The validity of the formulas used in the first version of the described method (Šimurda *et al.* 2021) was verified using a special software written in the Matlab live editor (available in Šimurda *et al.* 2021 as a Supplement S1_verification). We have verified the second version described in this paper in another Matlab file where resulting values of the essential elements of the electrical equivalent circuit were calculated using both versions for all measurements and the results were compared. This file is available on request.

Reference:

Šimurda, J., Šimurdová, M., Švecová, O., Bébarová, M. (2021). A new approach to the determination of tubular membrane capacitance: passive membrane electrical properties under reduced electrical conductivity of the extracellular solution.
bioRxiv preprint doi: https://doi.org/10.1101/2021.11.12.468264.

Supplemental Figure S1

Tyrode

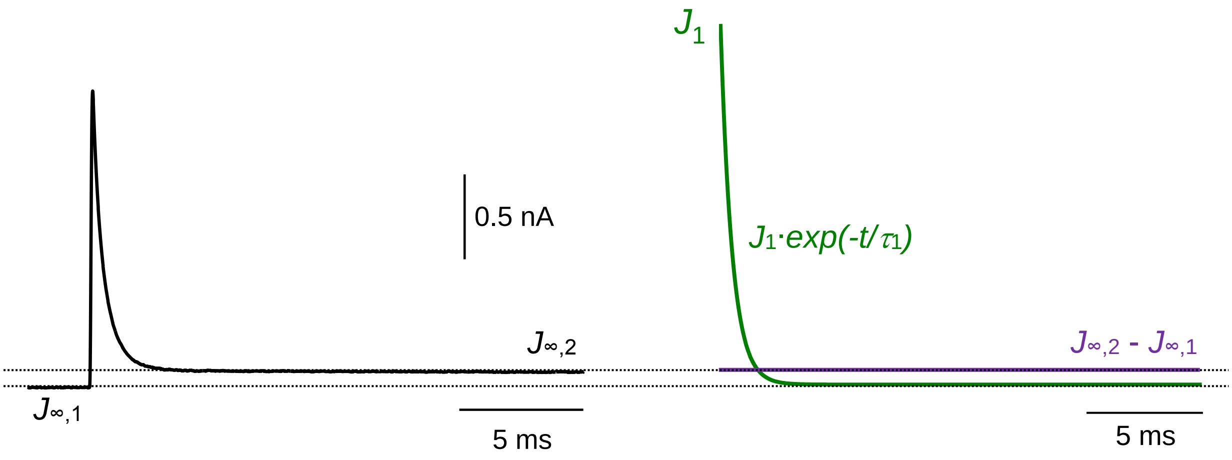

Resulting parameters				Calculated quantities	
J_1	τ_1 [ms]	$J_{\infty,1}$ [nA]	$J_{\infty,2}$ [nA]	$R_{a\ tyr}$ [MΩ]	C_{Tyr} [pF]
1.4940	0.4684	-0.011	0.0771	2.2249	229.6

Sucrose

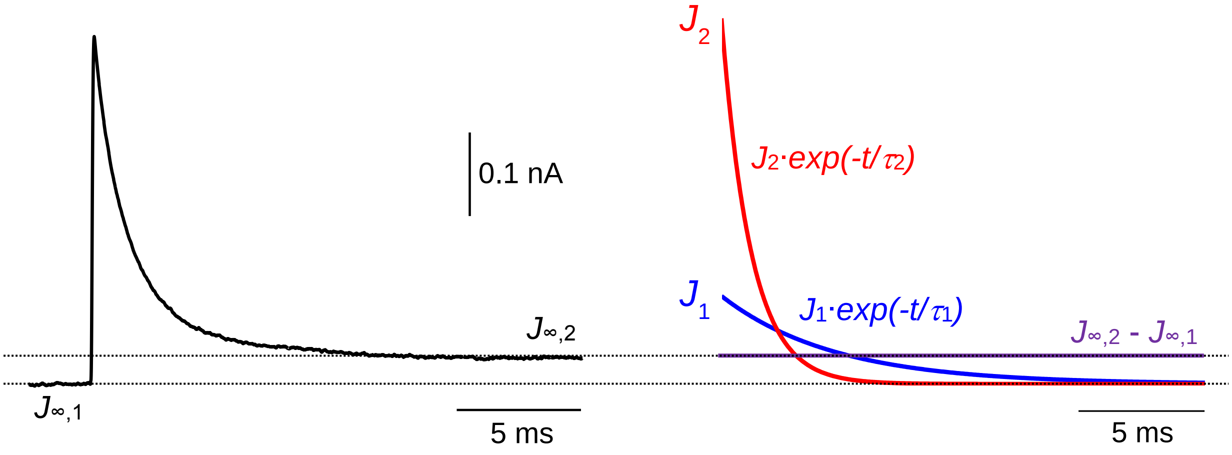

Resulting parameters					
J_1	τ_1 [ms]	J_2	τ_2 [ms]	$J_{\infty,1}$ [nA]	$J_{\infty,2}$ [nA]
0.0828	4.1389	0.2990	1.1342	0.7440	0.7741
**Calculated quantities**					
C_m [pF]	C_t [pF]	C_s [pF]	R_{a_suc} [MΩ]	f_t	
197.5	63.5	134.1	10.5308	0.3213	

Suppl. Fig. S1: Detail overview of the fitted curves including their parameters and the final computed parameters in Tyrode and sucrose solution in a representative cell from Fig. 1 in the main text.

Supplemental Figure S2

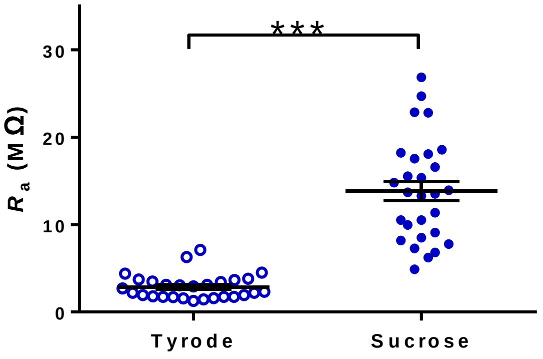

Suppl. Fig. S2: Access resistance R_a significantly increased in the presence of sucrose from 2.9 ± 0.3 MΩ to 13.8 ± 1.1 MΩ on average (n = 28, P < 0.001).

Supplemental Figure S3

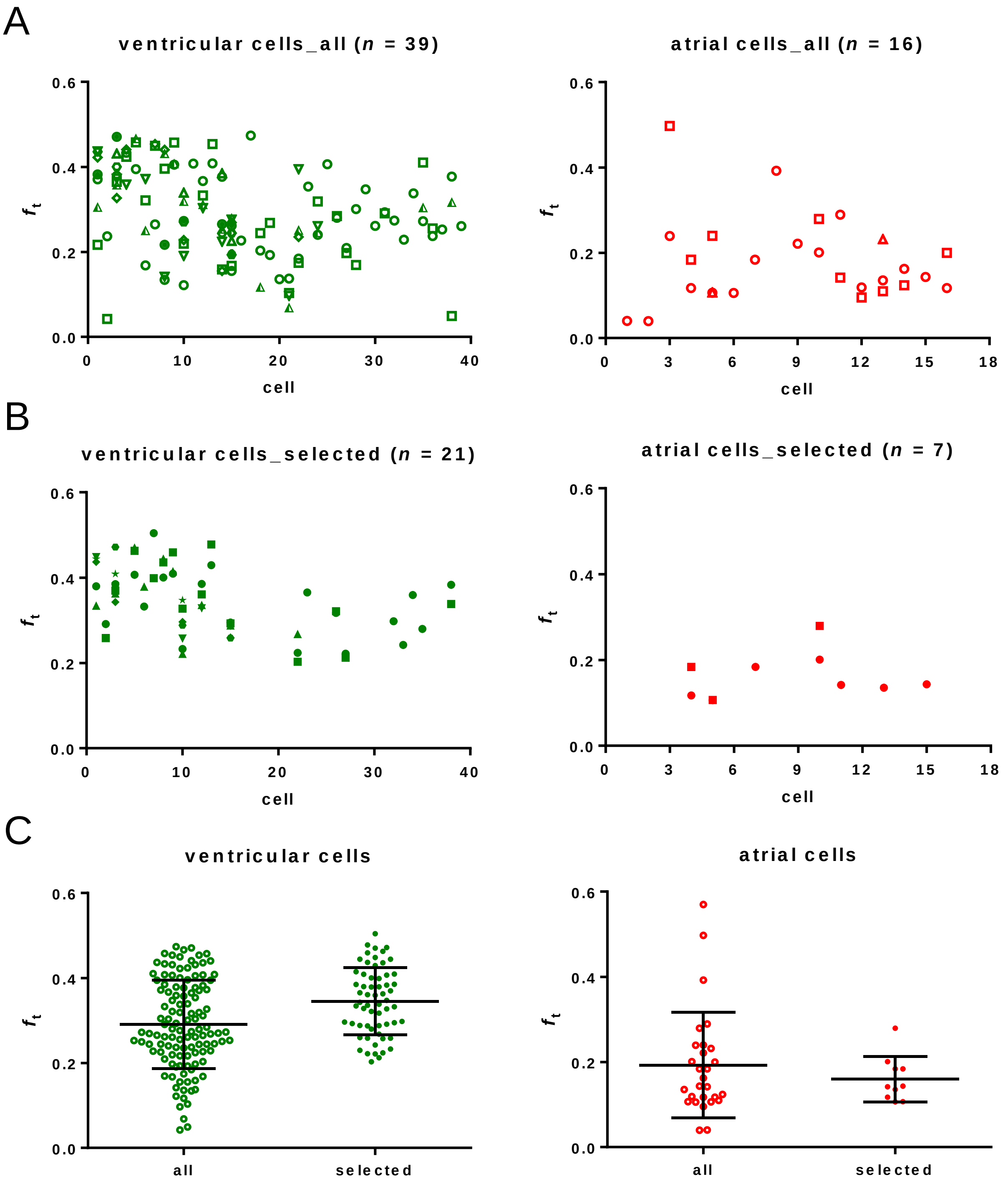

Suppl. Fig. S3: All performed measurements of the fraction of tubular membrane (f_t) and measurements selected based on the suggested strict criteria (left panels – ventricular myocytes, right panels – atrial myocytes). **A:** All f_t values (120 and 61 measurements out of 39 and 16 cells, respectively). **B:** Selected f_t values (28 and 10 measurements out of 21 and 7 cells, respectively). **C:** Comparison of all and selected f_t values including the average mean value ± SD; difference between the mean value in all and selected cells is not statistically significant in any case ($P > 0.05$ in both ventricular and atrial cells), however, variability of the data decreased after the selection procedure.

Supplemental Figure S4

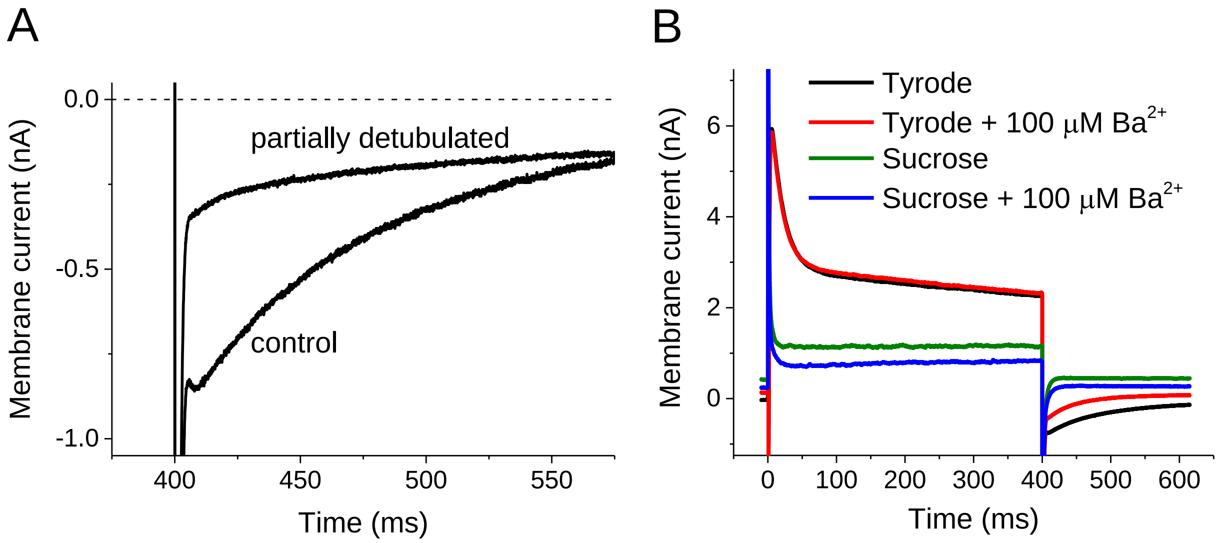

Suppl. Fig. S4: The tail membrane currents ($I_{K1,tail}$) generated in response to a repolarization step from +50 mV (400-ms pulse) to -75 mV. **A:** The effect of partial detubulation on $I_{K1,tail}$ (recorded in Tyrode solution in a representative cell that was treated with 225 μM imipramine for 15 min). **B:** $I_{K1,tail}$ recorded in Tyrode solution was sensitive to I_{K1}-inhibitor Ba^{2+} (100 μM). In sucrose solution, $I_{K1,tail}$ disappeared as observed in 8 measured cells (for explanation, see the main text).
